# Autologous micrografts from the palatal mucosa for bone regeneration in calvarial defects in rats: a radiological and histological analysis

**DOI:** 10.1186/s40729-020-00288-6

**Published:** 2021-01-25

**Authors:** Sawako Kawakami, Makoto Shiota, Kazuhiro Kon, Masahiro Shimogishi, Hajime Iijima, Shohei Kasugai

**Affiliations:** Department of Oral Implantology and Regenerative Dental Medicine, Tokyo Medical and Dental University, 1-5-45, Yushima, Bunkyo-ku, Tokyo, 113-8549 Japan

**Keywords:** Dissociated soft-tissue suspension, Bone regeneration, Palatal mucosa

## Abstract

**Background:**

The application of dental implants is often restricted by bone volume. In such cases, bone grafts are required, although bone graft materials have some disadvantages. Therefore, other effective approaches are needed. Our previous study showed that the autologous micrograft, a dissociated cell suspension made out of palatal connective tissue grafts, promoted bone-marrow cell proliferation and differentiation under osteogenic conditions. In this study, we aimed to evaluate the effects of dissociated soft-tissue suspensions relevant to bone regeneration in animal model.

**Material and methods:**

Twelve-week-old male Wistar rats were used in the study. Defects were created in rat calvaria, and were filled with hydrogel containing either dissociated soft-tissue suspension (test) or sucrose (control). The new bone formation was evaluated at 1 and 2 weeks after surgery (*n* = 16) by radiological and histological analysis.

**Results:**

The conducted radiological analysis showed that the new bone volume was significantly greater in the dissociated soft-tissue suspension group. This finding was further confirmed by the conducted histological analysis.

**Conclusions:**

The dissociated mucosa tissue suspension enhanced bone regeneration in vivo; thus, it is a promising potential method to aid the successful application for bone augmentation in the implant practice.

## Background

Dental implants are currently used as a standard treatment for patients with partial or complete edentulism [1, 2]. In fact, previous studies have reported a significant increase in patient satisfaction with the function, comfort, and aesthetics of implant prosthesis compared with removable dentures [3, 4]. Additionally, dental implants provide frequently long-term survival compared to conventional prostheses [5]. The long-term maintenance of implant function and aesthetics are followed; nevertheless, insufficient bone volume would restrict the placement of dental implant with adequate length and width [6].

Autograft (i.e., autologous bone grafts) is considered one of the most reliable bone augmentation methods [7] because it can provide the grafting of both osteogenic cells and essential growth factors that are required for bone healing and remodeling [8]. However, the volume of autologous bone that can be harvested to support dental implants is limited [9], and furthermore, autologous bone harvesting may incur injuries to nerves, arteries, and/or adjacent teeth [7]. Conversely, the use of allografts or xenografts for bone augmentation does not require the patient to undergo additional surgery; however, it cannot provide enough osteogenic properties [10], and can induce an immunogenic reaction [8]. Thus, novel methods are urgently needed to facilitate effective and safe bone augmentation in the clinical situation.

Recently, autologous micrograft, which is a dissociated cell suspension made out of tissue grafts, has received much attention as a promising material for soft-tissue engineering applications and plastic surgical procedures in the clinical setting [11]. Autologous micrograft (diameter < 50 μm) has been shown to facilitate improved wound healing [12] and hair transplantation [13], likely by ensuring the transplantation of an optimal cellular microenvironment and extracellular matrix, as well as of the target cells [14].

Several studies have demonstrated that fibroblast growth factors (FGF) and vascular endothelial growth factor (VEGF), which mediate bone regeneration, are released from mucosal tissues [11, 15–17]. Furthermore, other studies have suggested that dissociated soft-tissue suspension enriched for progenitor cells can enhance tissue regeneration [13, 18]. Based on these findings, we hypothesized that dissociated soft-tissue suspension likely contains growth factors capable of enhancing osteogenesis. In fact, we successfully demonstrated that dissociated soft-tissue suspension enhanced bone-marrow cell proliferation, osteogenic differentiation, and mineralized nodule formation in vitro study [19]. Furthermore, we suggested that the efficacy of bone regeneration using dissociated soft-tissue suspension with the collagen membrane as a scaffold in vivo study [20]. It indicated that dissociated soft-tissue suspension with collagen membrane promoted bone healing in rat calvaria defect model. However, the collagen membrane is biomaterial, so that biological effects might be reflected in the result. In addition, the application of biomaterial may have possibility for allergy [21]. Therefore, we selected the synthetic peptide hydrogel as a scaffold to avoid the biological effect and other complication in the present study. The aim of this study was to evaluate the potential of dissociated soft-tissue suspension itself generated from palatal mucosa to enhance bone regeneration in rat calvarial bone graft model.

## Materials and methods

### Preparation of dissociated soft-tissue suspension

**Fig. 1:**
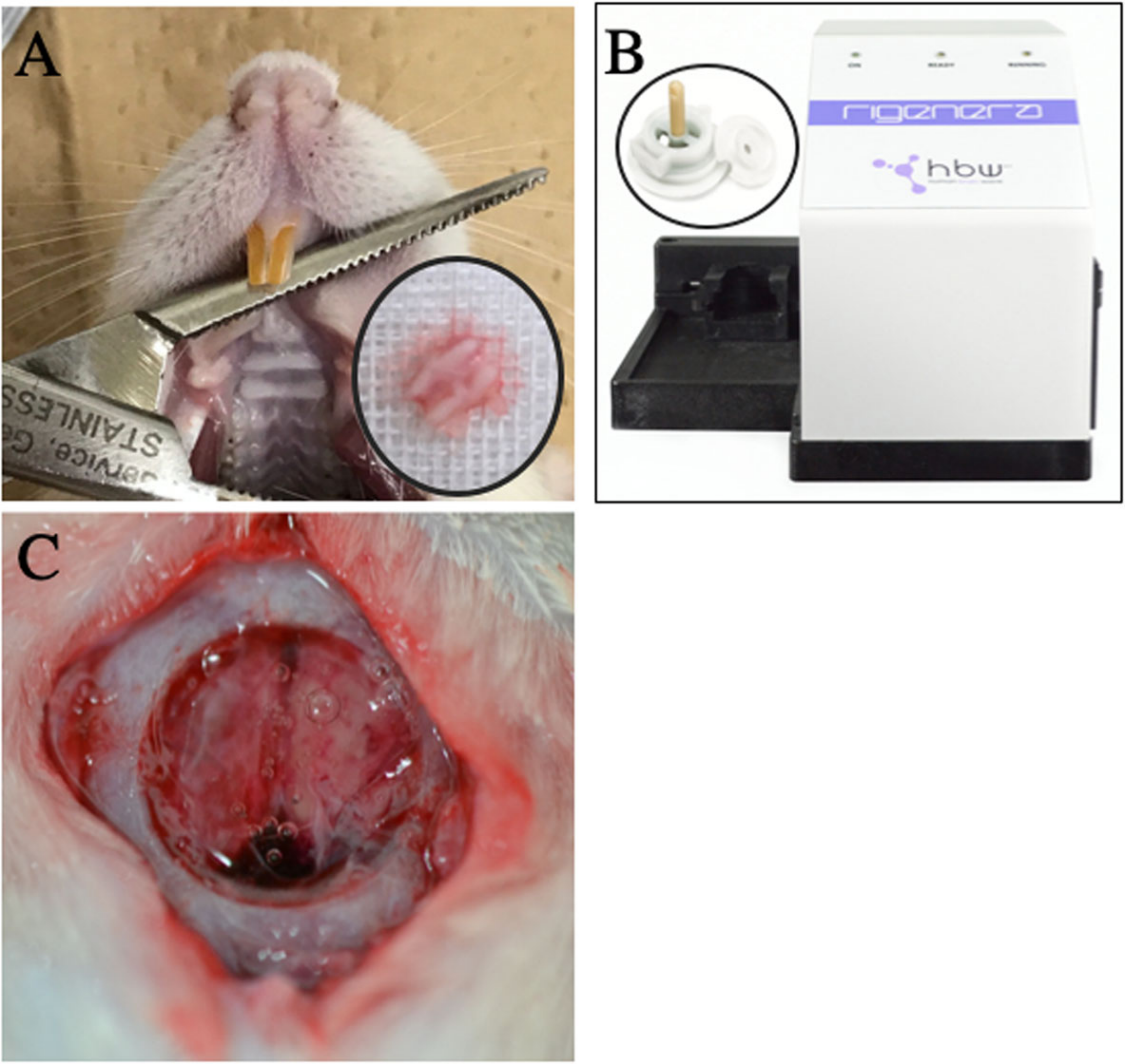
Preparation of dissociated soft-tissue suspension. **a** Harvesting of rat palatal mucosa. **b** Use of the tissue dissociative machine (Rigenera; Human Brain Wave, Italy). **c** The dissociated soft-tissue suspension was mixed with self-assembling peptide and placed into the calvarial defect

The autologous micrograft, which is a dissociated cell suspension made out of palatal connective tissue grafts, was prepared following procedure. Twelve-week-old male Wistar/ST rats (body weight, 340–370 g) were used after acclimatization (2 weeks). The rats were kept in cages at constant temperature, with a 24-h light-dark cycle, and provided ad libitum access to food and water during the experiment period. Rats were randomly allocated into two (‘test’ and ‘control’) groups, and anesthetized via initial inhalation of 4% (v/v) isoflurane for 2 min, followed by an intramuscular injection of ketamine (84 mg/kg Ketalar; Daiichi Sankyo Inc., Tokyo, Japan) and xylazine (12 mg/kg; Selactar; Bayer Yakuhin Ltd., Osaka, Japan). A partial thickness palatal mucosa section (2 mm × 2 mm) was then resected from each rat. The superficial epithelium was removed from each mucosa, and the remaining mucosa was cut in half (Fig. 1a). This tissue graft was immediately processed into a dissociated soft-tissue suspension with 1 mL of 10% sucrose solution using a tissue dissociative device (Rigenera; Human Brain Wave, Italy, Fig. 1b) for 60 s [11].

### Surgical procedures

After harvesting dissociated soft-tissue suspension, the calvarial bone of each rat (*n* = 16) was exposed via a linear skin incision (producing a skin flap), and the periosteum was raised. A full-thickness 8-mm-diameter bone defect was created in the center of the exposed calvaria using a bone trephine bur. In the test group, the dissociated soft-tissue suspension made from autograft was mixed with self-assembling peptide hydrogel (Corning PuraMatrix; Thermo Fisher), and then placed into the calvarial defect. In the control group, a 10% sucrose solution was combined with hydrogel and then placed into the calvarial defect (Fig. 1c). The skin flap was then repositioned and sutured with 4-0 nylon. The rats were sacrificed at 1 and 2 weeks (*n* = 4/group) after surgery.

### Radiographic analysis

Samples were subjected to a quantitative radiological analysis using a micro-computed tomography scanner (100 kV, 30 μA, SMX-90CT; Shimadzu Corporation, Kyoto, Japan). The new bone volume (BV) and the ratio of new bone volume relative to the total tissue volume (new bone volume/total tissue volume; BV/TV) and the ratio of new bone area were assessed in this study. The region of interest was precisely positioned over each calvarial defect, determined via histological analysis. BV indicated that the newly formed bone including mature and immature bone volume, and TV indicated that BV and the newly formed soft tissue volume. BV and TV were calculated by counting the number of radiopaque voxels at the site using TRI/3D BON software (RATOC System Engineering Co., Ltd., Tokyo, Japan) within the region of interest. After reconstruction, the new bone and soft tissue were segmented using a threshold 396.58125 mgHA/cm^3^ and 235.33078 mgHA/cm^3^, respectively, according to histological analysis and literatures [22, 23]. The ratio of new bone area was evaluated using the three-dimensional (3D) images observed from vertical side were binarized using ImageJ 1.50i (Wayne Rasband, National Institutes of Health, USA) and calculated the bone fill area within the defect area.

### Histological analysis

Samples were immediately fixed for 14 days in neutral 10% formalin, and decalcified for 3 weeks in 10% formic acid (room temperature). The specimens were then trimmed down to an area immediately surrounding the calvarial defect, dehydrated with ethanol, embedded in paraffin, sectioned (5 μm) using a microtome, and stained with hematoxylin and eosin. The center of the sample was observed using an optical microscope (BIO ZERO BZ-8000; Keyence).

### Statistical analysis

All data were examined for normality by Shapiro-Wilk tests; however, since not all data achieved normality, comparisons were made using Mann-Whitney *U* test with SPSS (SPSS version 22, IBM). A *p* value *<* 0.05 was considered to indicate statistical significance.

## Results

### Volumetric radiological analysis of test- and control-group animals

**Fig. 2:**
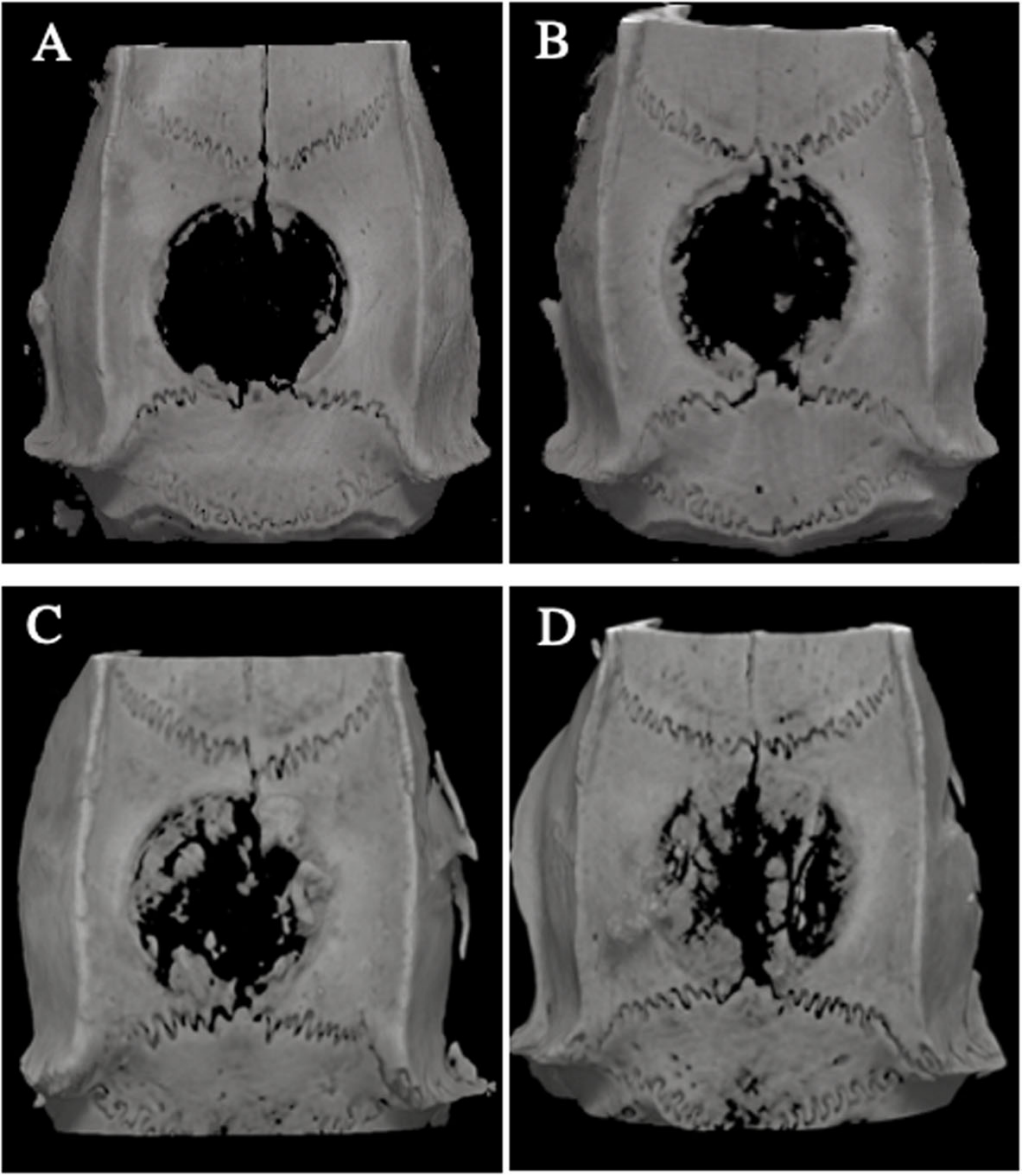
Radiographic analysis was performed. **a** 3D images at 1 week in the control. **b** 3D images at 1 week in the test. **c** 3D images at 2 weeks in the control. **d** 3D images at 2 weeks in the test

**Fig. 3:**
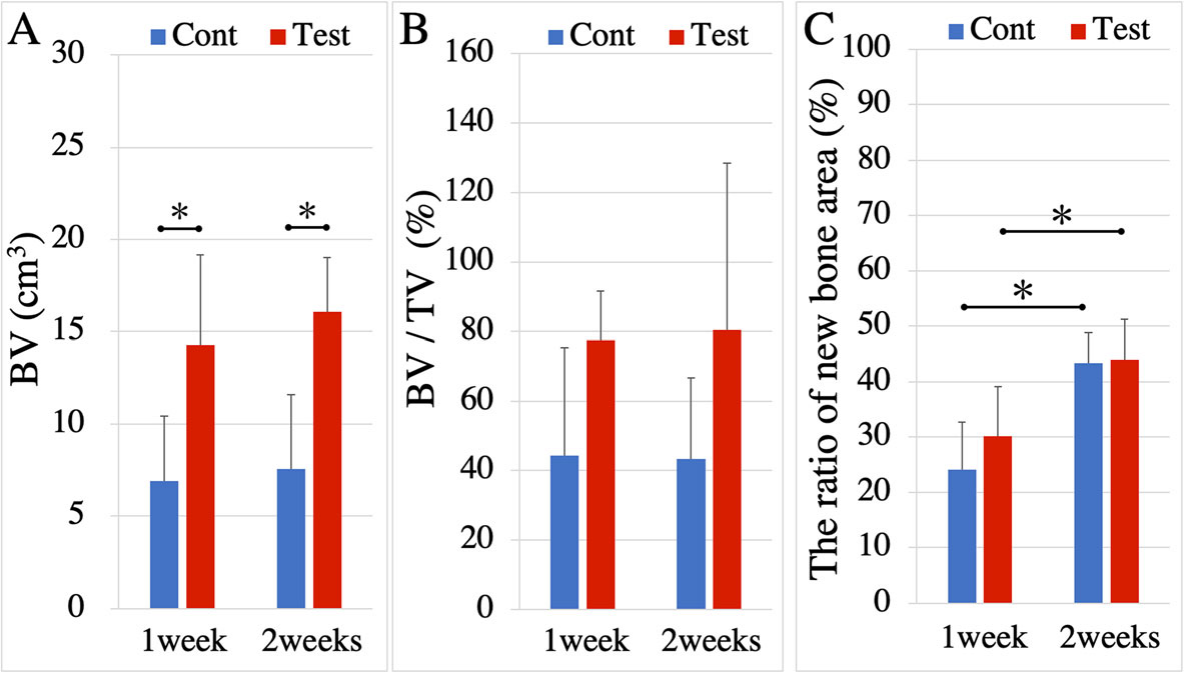
BV, BV/TV, and the ratio of new bone area change in each group. **a** BV were shown and significant differences were found. **b** BV/TV were shown and no significant differences were found between groups at the same time points. **c** The ratio of new bone area change were shown and no significant differences were found. *p < 0.05

New bone in the calvarial defects of 3D images is shown in Fig. 2. BV was significantly greater in the test group than in the control group at both analyzed time points (*p* = 0.024 and *p* = 0.024 at 1 week and 2 weeks between groups, respectively; Fig. 3a). The BV/TV was not significantly different between the test and control groups (*p* = 0.114 and *p* = 0.200 at 1 week and 2 weeks between groups, respectively; Fig. 3b). In contrast, the ratio of new bone area was significantly increased in each group between 1 and 2 weeks (*p =* 0.024 and *p* = 0.024 in the control and test groups between 1 and 2 weeks, respectively), but was not significantly different between the test and control groups (*p* = 0.686 and *p* = 1.000 at 1 week and 2 weeks between groups, respectively; Fig. 3c).

### Histological observations of test- and control-group animals

**Fig. 4:**
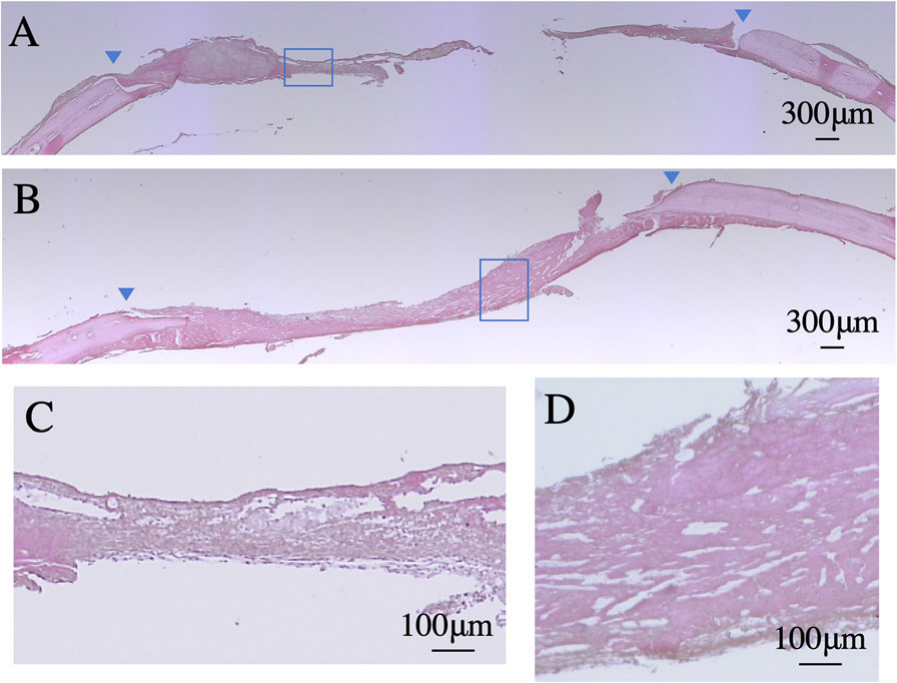
Histological images of decalcified sections stained with hematoxylin-eosin at 1 week. **a** In the control. **b** In the test. Blue arrows show the original defect margins. **c** High magnification image of in flamed area in the control. **d** High magnification image of in flamed area in the test. The center of the sample was observed

**Fig. 5:**
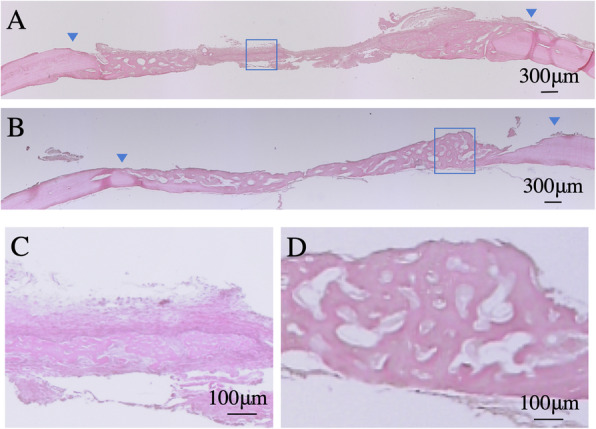
Histological images of decalcified sections stained with hematoxylin-eosin at 2 weeks. **a** In the control. **b** In the test. Blue arrows show the original defect margins. **c** High magnification image of in flamed area in the control. **d** High magnification image of in flamed area in the test. The center of the sample was observed

Histological images of the incurred calvarial defects are shown in Fig. 4 and Fig. 5. In the control group, most calvarial defects were covered with loose connective tissue by 1 week after surgery (Fig. 4a). At this time point, fibrous connective tissue (including cells with flat nuclei) was observed in parallel along the anteroposterior axis of the defect margin (Fig. 4c). Immature bone formation with lacunae extended from the margin toward the center of the defect. Some relatively large noncellular structures were preserved; however, the disrupted section of the noncellular structure was surrounded by cell-rich fibrous connective tissue.

In contrast, in the test group of 1 week, exhibited newly formed bone with large lacunae that extended from the margin toward the center of the defect (Fig. 4b). Immature cell-rich connective tissue that stained strongly for eosin, and that included both a fine network and infinite lacunae, extended parallel to the parietal bone throughout the inner part of the defect, and was contiguous with immature bone (Fig. 4d). The noncellular structure was better retained at the upper part of defects, and more blood vessels were observed in the test group than in the control group.

At 2 weeks after surgery, the control group exhibited newly formed bone with large lacunae near the defect margin (Fig. 5a). The staining pattern exhibited by the new bone was equivalent to that of host bone. Immature loose connective tissue covered the entire upper section and the center of the inner section of the defect. New bone formation was observed adjacent to the immature fibrous connective tissue at the lower section of the defect (Fig. 5c). Part of the noncellular structure was retained.

In the test group of 2 weeks, the newly formed bone with lacunae of various sizes (some of which contained cells within their inner spaces) were located close to the margin of the defect (Fig. 5b). Notably, a much larger amount of new bone was observed in the test group compared with that to the control group. Lining cells were observed surrounding the newly formed bone. The defect margins were unclear because they exhibited the same staining as the host bone; however, bone lamellae were observed irregularly (Fig. 5d). Immature connective tissue and noncellular structures were not retained at the margin of defect.

## Discussion

In this study, we investigated the efficacy of a dissociated soft-tissue suspension for bone augmentation. In recent years, enamel matrix derivatives (EMDs), and growth factors such as bone morphogenetic proteins (BMPs), have been used to augment bone formation in the clinical situation; however, EMDs have been shown to produce different effects in various osteoblast types, likely as a result of missing growth factors [24]. Similarly, the use of BMPs has been reported to cause edema, tissue damage, and/or teratogenesis [25]. Thus, novel and effective approaches to facilitate successful bone augmentation are required.

The conducted radiographic analysis identified new bone in all of the analyzed samples; the BV was significantly higher in the test group than in the control group at both of 1 and 2 weeks. In contrast, no significant differences were found in the ratio of new bone area between the control and test groups. At the histological observation in the control group, thickness of newly formed bone was less than the test group. These results showed that trabecular structure formation was increased in the test group compared to the control group at 1 and 2 weeks after surgery. This finding is consistent with our previously conducted in vivo study, which also revealed a significant increase in the BV that was formed after 1 week of exposure to a palatal mucosa suspension. The BV/TV in the present study was not significantly different between the test and control groups; although, the BV was significantly higher in the test than the control group. It suggests that the generated dissociated palatal mucosa suspension enhanced not only new bone formation but soft tissue formation such as fibrous tissue at the incurred defects.

The conducted radiographic and histological analyses also confirmed new bone formation in all analyzed samples. Although hydrogel was retained in the defects in both groups after 1 week, it was observed in larger quantities in the control group than in the test group, suggesting that the rate of hydrogel disruption was increased in the test group compared to that of the control group. In addition, cell-rich connective tissue was observed only in the test group, suggesting that the dissociated soft-tissue suspension facilitated its formation by enabling cell assembly. At 2 weeks after surgery, newly formed bone with irregular lamellae structure was observed throughout the test defects. Furthermore, bone-lining cells were observed on the newly formed bone in the test group. Nevertheless, together, these findings support that the dissociated soft-tissue suspension promoted osteogenesis and bone maturation.

In our previous study, we observed newly formed bone at the margin of incurred defects after they were treated with a collagen membrane micrograft group for 1 week [20]. In contrast, the current study showed newly formed bone and surrounding connective tissue located adjacent to the host bone at the margin of the incurred defects. Moreover, although the border between the host and newly formed bone became unclear at 4 weeks after surgery in our previous study [20], the current study showed that the newly formed bone was connected to the host bone by 2 weeks after surgery. These findings suggest that the processes of bone maturation differ between the two studies. In fact, the self-assembling peptide matrices used in the current study comprised a synthetic and resorbable hydrogel that was used for the delivery of therapeutics during preclinical research [26]. The gradual absorption of the self-assembling peptide matrices is considered to facilitate the sustained release of mucosal tissue delivered signaling molecules and growth factors from the dissociated soft-tissue suspension and to support bone regeneration to a greater extent than the previously developed collagen micrograft, and enabling a significant increase the new bone volume in this study at 2 weeks in the test group.

The dissociated soft-tissue suspension generated in this study was produced from rat palatal mucosa. Some previous studies have demonstrated that mucosal tissues express high levels of pro-angiogenic factors, such as FGF2 and FGF8, which promote osteoblast differentiation and bone formation [11, 15, 16]. Moreover, exposure to dissociated mucosal tissue has been shown to promote VEGF expression [16], and thereby stimulate wound healing via multiple mechanisms, including collagen deposition, angiogenesis, and epithelization [27]. VEGF-enhanced angiogenesis has also been shown to improve bone regeneration [17]; thus, in the present study, we harvested and generated the dissociated soft-tissue suspension from a relatively large amount of palatal tissue. Further studies are required to investigate which specific pro-osteogenic growth factors and signaling molecules. Similarly, while d’Aquino et al. [28] previously showed that micrograft produced using the herein employed dissociation device promote cell viability, further studies are needed to evaluate and confirm the effects of the generated palatal mucosa-derived dissociated soft-tissue suspension on cell viability.

Autogenous tissue graft does not incur any risk of immunogenic reaction or latent infection. The dissociated soft-tissue suspension developed in the current study promises to improve the rate at which autografts are currently used to achieve bone regeneration, and may also reduce patient treatment and recovery periods. Continued research is needed to assess its potential as a local injection or adjuvant for use in combination with scaffold material to repair bone defects in the clinical setting.

## Conclusions

The use of dissociated palatal mucosa tissue suspension generated by autogenous tissue promoted bone formation in this animal model. The appreciation of dissociated soft-tissue suspension for bone augmentation promises to improve the patient burden, which is less amount of harvesting autograft than conventional bone graft in the clinical situation. Future study needs to investigate the scaffold which was more effective material for bone augmentation.

## Data Availability

The datasets used in the study are available from the corresponding author on reasonable request.

## Abbreviations

FGF: Fibroblast growth factors
VEGF: Vascular endothelial growth factor
BV: New bone volume
TV: Total tissue volume
3D: Three-dimensional
EMDs: Enamel matrix derivatives
BMPs: Bone morphogenetic proteins
